# Morpho-Physiological Variation of White Spruce Seedlings from Various Seed Sources and Implications for Deployment under Climate Change

**DOI:** 10.3389/fpls.2016.01450

**Published:** 2016-09-30

**Authors:** Isabelle Villeneuve, Mohammed S. Lamhamedi, Lahcen Benomar, André Rainville, Josianne DeBlois, Jean Beaulieu, Jean Bousquet, Marie-Claude Lambert, Hank Margolis

**Affiliations:** ^1^Centre d’Étude de la Forêt, Faculté de Foresterie, de Géographie et de Géomatique, Université Laval, QuébecQC, Canada; ^2^Direction de la Recherche Forestière, Ministère des Forêts, de la Faune et des Parcs, QuébecQC, Canada; ^3^Canadian Wood Fibre Centre, Canadian Forest Service, Natural Resources Canada, QuébecQC, Canada

**Keywords:** white spruce, climate change, assisted migration, seed orchard

## Abstract

Because of changes in climatic conditions, tree seeds originating from breeding programs may no longer be suited to sites where they are currently sent. As a consequence, new seed zones may have to be delineated. Assisted migration consists of transferring seed sources that match the future climatic conditions to which they are currently adapted. It represents a strategy that could be used to mitigate the potential negative consequences of climate change on forest productivity. Decisions with regard to the choice of the most appropriate seed sources have to rely on appropriate knowledge of morpho-physiological responses of trees. To meet this goal, white spruce (*Picea glauca* [Moench] Voss) seedlings from eight seed orchards were evaluated during two years in a forest nursery, and at the end of the first growing season on three plantation sites located in different bioclimatic domains in Quebec. The morpho-physiological responses obtained at the end of the second growing season (2+0) in the nursery made it possible to cluster the orchards into three distinct groups. Modeling growth curves of these different groups showed that the height growth of seedlings from the second-generation and southern first-generation seed orchards was significantly higher than that of those from other orchards, by at least 6%. A multiple regression model with three climatic variables (average growing season temperature, average July temperature, length of the growing season) showed that the final height of seedlings (2+0) from the first-generation seed orchards was significantly related to the local climatic conditions at the orchard sites of origin where parental trees from surrounding natural populations were sampled to provide grafts for orchard establishment. Seedling height growth was significantly affected by both seed source origins and planting sites, but the relative ranking of the different seed sources was maintained regardless of reforestation site. This knowledge could be used, in conjunction with transfer models, to refine operational seed transfer rules and select the most suitable sites in an assisted migration strategy.

## Introduction

Current climate conditions are evolving at a rapid pace and the majority of species must adapt *in situ* to new climatic conditions or migrate toward conditions that are more favorable to their growth (Aitken and Whitlock, 2013; Alberto et al., 2013; GIEC, 2013). However, with their relatively long life cycle and their limited natural migration capacity, populations of tree species may not be able to adapt to anticipated future climatic conditions, resulting in local maladaptation and a reduction in forest productivity (Carter, 1996; Iverson et al., 2004; Andalo et al., 2005; Beaulieu and Rainville, 2005).

Plantations represent 7% of the area occupied by forests worldwide and they are recognized as a complement to natural forests for wood supply and as a tool for adaptation and mitigation in the face of climate change (FAO, 2010). However, to assure plantation success, the optimal adaptation of genetically improved stock and that from natural populations must be taken into consideration in reforestation programs (Andalo et al., 2005; Beaulieu and Rainville, 2005; Lu et al., 2014). In many Canadian provinces, the transfer of seed sources has been revised and in certain cases, has been modified in the view of adaptation to anticipated climate change (Pedlar et al., 2011). For example, in British Columbia, it is now possible to transfer seed sources upward an additional 200 m in elevation for the majority of commercial species (O’Neill et al., 2008). The different migration strategies are mainly based on empirical models (Beaulieu et al., 2004; Thomson and Parker, 2008), but risks are nevertheless still difficult to assess in particular due to uncertainty related to global warming predictions.

In recent years, about 130 million seedlings have been used for reforestation in Québec and around 20% of these are white spruce (*Picea glauca* [Moench] Voss). In terms of its climatic distribution, white spruce is a species whose natural range extends east to west across continental North America from the tree line to the northern United States (Nienstaedt and Zasada, 1990). The species harbors extensive genetic variation in quantitative traits compared to many other commercial conifer species (Beaulieu et al., 2009), which is in part related to geography and climatic gradients (Li et al., 1997; Andalo et al., 2005). Genetic variation at many genes has further been shown to be tightly related to climatic factors (Hornoy et al., 2015). In 2012, 98% of the seeds used to produce white spruce seedlings were from improved sources (first- and second-generation seed orchards) that exhibited a genetic gain of 10 to 15% in height compared to unimproved plantation stock (Rainville et al., 2003; Beaulieu et al., 2009). The production of multiple clones by somatic embryogenesis is currently integrated into the operational chain for rooted cuttings and processes for their characterization and selection have been developed both in nurseries (Lamhamedi et al., 2000; Wahid et al., 2012a) and on reforestation sites (Grossnickle, 2000; Lamhamedi and Gravel-Grenier, 2012; Wahid et al., 2012b, 2013). Association genetics and genomic selection approaches have also been developed for different traits related to growth and wood quality of this species (Beaulieu et al., 2011, 2014a,b).

First-generation seed orchards established in Québec in the 1980s were made up of grafted plus-trees selected in local natural stands. Thus, each seed orchard provides genetically improved seeds for a boundary-fixed seed zone. Seed transfer guidelines were later established using growth measurements and geographical coordinates of seed sources collected in provenance-progeny tests established in the 1960-1970s (Li et al., 1997). A transfer model was later developed to include climatic variables (Andalo et al., 2005; Beaulieu and Rainville, 2005) and regional circulation models and scenarios (Rainville et al., 2014) to predict plantation yield under changing climatic conditions. Even though the transfer model can guide the selection of best adapted seed sources to future climate conditions with the aim of maximizing adaptation and productivity of plantations and help refine current seed zones, it is important to better understand the parameters responsible for the differential adaptation of white spruce seed sources used in reforestation programs. Hence, morpho-physiological characterization in nurseries and on the planting sites simulating assisted migration scenarios should provide better knowledge about the response of the different sources with respect to biotic and abiotic stresses. This knowledge could be used in conjunction with seed transfer models to refine the selection of genetic material that is both highly productive and tolerant in the face of rapidly changing climates, and potentially help delineate new seed zones. This study is the third in a series in which the ecophysiological related-traits of eight white spruce seed orchards were evaluated. The two previous studies revealed, for seedlings grown under greenhouse conditions (Benomar et al., 2015) and site plantations (Benomar et al., 2016), the presence of local adaptation of these populations to their biophysical environments which suggest the existence of adaptative ecophysiological divergence.

The general goal of this study was to assess the impacts of climate change on the adaptation of white spruce seed sources based on morpho-physiological traits. The first evaluation was made during two consecutive years in a forest nursery located within the natural distribution range of the species. The following year, seedlings were planted on three sites representative of Quebec’s three major bioclimatic domains and of the natural ecological gradient where white spruce is reforested, thus simulating assisted migration; the second evaluation was made after the first growing season on the field. Seedlings from eight different genetic seed sources were used: six first-generation seed orchards representative of local sources and two second-generation seed orchards. This characterization allowed us to determine morpho-physiological differentiation among the seed sources and to establish links between seedling traits under forest nursery conditions and the climatic conditions at the location of the seed orchards where parental trees were selected to provide grafts for orchard establishment. Thus, the objectives of the study were: (i) to examine the variation in morpho-physiological traits among white spruce seed sources over two consecutive growing seasons (1+0 and 2+0) in a forest nursery and (ii) to evaluate the growth and survival of seed sources on forest sites during the seedling establishment phase in response to a simulated assisted migration.

## Materials and Methods

### Origin of the Genetic Material

**FIGURE 1:**
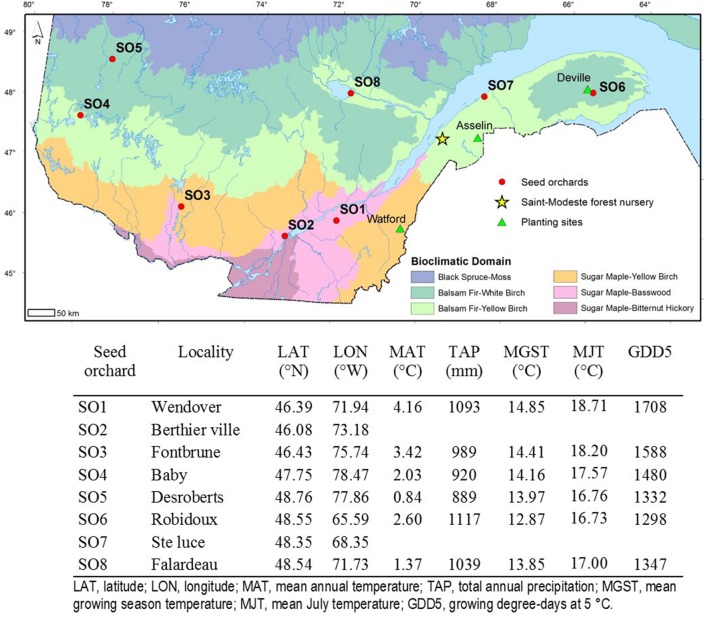
**Identification, location and geographic coordinates of the eight white spruce seed orchards in the province of Quebec as well as the location of the Saint-Modeste forest tree nursery where the seedlings were raised for their first two growing seasons.** The orchards are identified from 1 to 8, in order of increasing latitude. Geoclimatic data of the centroid of selected trees from local provenances that make up the first-generation white spruce seed orchards were also indicated. Climatic data for the second-generation seed orchards (SO2 and SO7) are not determined because they are composed of trees representing multiple provenances from the province of Québec and Ontario. A description of bioclimatic domains can be found in Saucier et al. (2010).

White spruce seedlings used in this study originated from six first-generation and two second-generation seed orchards. The seed orchards were chosen in accordance with their contribution and use in the reforestation program of the Ministère des Forêts, de la Faune et des Parcs of Québec. The first-generation seed orchards were established for each reforestation region using grafts of plus-trees (Superior phenotypes to the surrounding trees in the stand) selected from local natural stands (Beaulieu et al., 2009). Thus, each of these orchards must be considered as a representative of the surrounding local natural population. Taken together, they were representative of a territory located between 45°N and 50°N of latitude and between 57° and 79°W of longitude. The second-generation seed orchards were established using grafts of plus-trees selected in the top-performing open-pollinated families assessed in a range-wide provenance-progeny test replicated on eight sites. These superior families are from across Québec and Ontario. These seed orchards provide genetically improved seed for the reforestation of two major zones in Québec, respectively, located south and north of approximately 47°N latitude (Li et al., 1997), and representing the sugar maple and the balsam fir bioclimatic domains, respectively. The orchards selected for this study were numbered 1 to 8 (SO1 to SO8), according to their increasing latitude of location (**Figure 1**), with numbers 2 and 7 corresponding to second-generation orchards (**Figure 1**).

### Seedling Nursery Production of the Different Seed Sources (1+0 and 2+0)

For each of the eight white spruce seed orchards, seed was extracted from cones harvested over two years to ensure a better contribution of the different clones and to account for the inter-annual variation in cone production. To avoid a large difference in seed size and variations in seedling height growth related to seed dimensions (Tousignant et al., 2008), only seeds with a diameter ≥1.75 mm were sown. This screening of seeds according to their size is used operationally in the province of Québec in an effort to homogenize production and avoid undesirable maternal effects. Three seeds per cavity were manually sown into 25-310 containers (25 cavities per container, 310 cm^3^ per cavity; *IPL*^®^, Saint-Damien, Bellechasse, QC, Canada) on May 10 and 11, 2011, at the Saint-Modeste forest nursery (47°50′N, 69°23′W). The containers were filled with peat/vermiculite substrate (v/v, 3/1) with a density of 0.10 g cm^-3^. A total of 125 containers were seeded for each of the eight orchards, for a total production of 25,000 plants.

The seeded containers were placed in a white polyethylene-covered tunnel and were raised approximately 20 cm above the ground surface. To avoid any differential growth among the different seed sources due to the possible presence of an environmental gradient in the tunnel, each seed orchard was represented by 125 containers, which were arranged in five randomized complete blocks. Twenty-five contiguous containers per seed orchard were allocated to each block. The seed orchards were randomly distributed within each block. After germination, seedlings were thinned to one seedling/cavity on mid-July 15, 2011. During the first (1+0) and the second growing season (2+0) (the first number indicates the number of years the seedlings were raised from seeds in the container. The second refers to the number of years the seedling were raised in a plantation), seedlings were irrigated and fertilized using a mechanized boom (Aquaboom, Industrie Harnois, Saint-Thomas-de-Joliette, Québec) in order to minimize the spatial variability of substrate water content (Lamhamedi et al., 2006). The coefficient of uniformity of the boom was between 95 and 98%, indicating that water was uniformly distributed over the containers (Lamhamedi et al., 2001). The substrate water content was adjusted in accordance with seedling growing stage during the first growing season (1+0) (Lamhamedi et al., 2001), then maintained between 40 and 45% (v/v) during the second growing season (2+0) (Lamhamedi et al., 2006).

The tunnel was covered with milk-white polyethylene, 100 μm thick, which transmitted 50 to 55% of incident light (Ginegar Plastic Products Ltd, Multi-layer greenhouse cover film, type UVA/White 45%). The cover could be retracted along both sides to facilitate aeration and modify the air temperature inside the tunnel. During the germination and active growing periods (May to late July), mean air temperature varied between 13 and 29°C. During the period of bud formation and natural hardening (early August to late September), the average daily temperature inside the tunnel decreased progressively, varying from 23 to 6°C. Relative humidity fluctuated between 60 and 87%.

During the week of December 19, 2011, the seedlings were transferred outside an unheated tunnel, where they spent the winter under a cover of natural snow. The original experimental design was maintained throughout this period. During the second growing season (2+0), the seedlings were cultivated outside under natural environmental conditions.

The fertilization regime was adjusted bi-weekly during both growing seasons (1+0 and 2+0) using the PLANTEC software (Girard et al., 2001). At the end of the first growing season (1+0), each seedling had received 71 mg of N (40 mg N-NH_4_, 28 mg N-NO_3_, 2 mg N-Urea), 20 mg of P, 31 mg of K and 7 mg of Mg as well as micronutrients (Mn, Cu, Fe and B). Fertilizers were applied in accordance with the growing stage of the seedlings between June 9 and September 22, 2011. At the end of the second growing season (2+0), in addition to micronutrients, each seedling received, between May 4 and October 4, 2012: 222 mg of N (91 mg N-NH_4_, 125 mg N-NO_3_, 6.5 mg N-Urea), 58 mg of P, 83 mg of K, 57 mg of Ca, and 0.9 mg of Mg.

### Growth and Mineral Nutrition Variables 1+0 and 2+0

Destructive sampling was conducted bi-weekly between August 2 and October 26, 2011 (seven sampling dates) during the first growing season and between May 16 and October 17, 2012 (12 sampling dates) during the second growing season. On each sampling date, five seedlings per orchard were randomly selected from each of the five blocks, for a total of 200 seedlings/sampling date. The five seedlings/orchard/block were harvested from the same container. The choice of the first container selected on each sampling date as well as the choice of the five cavities in this container were determined randomly, whereas the containers for the subsequent orchards and blocks were selected systematically given that their relative position in the block was the same as the first orchard’s initial container. The containers that were sampled during the first season were not part of the random selection process in the second season. The cavities harvested during the sampling process were filled with peat moss and covered in silica to eliminate a bias in the root growth of the seedlings in the neighboring cavities. After each harvest, the seedlings were cleaned with compressed air and washed to remove all traces of substrate from their roots. The five seedlings harvested from the same container were wrapped in wet paper and placed in plastic bags identified by orchard and by block. They were shipped to the QMFWP labs the same day and maintained under refrigerated conditions until they were prepared for drying.

Height (terminal bud to collar) and root collar diameter measurements were made within 24 to 48 h of harvesting. Once the measurements were completed, each seedling was cut off at the root collar and the shoot (above ground) and root system were placed in separate paper bags and oven-dried for a minimum of 48h at 65°C. Then the shoot and root dry mass were determined for each of the 200 seedlings sampled.

For each sampling date over the two growing seasons, analysis of the mineral nutrition (N, P, K, Ca, Mg) of the shoot and root tissues was performed on composite samples (five seedlings/orchard/block). The total nitrogen concentration was determined by combustion of the sample at high temperature by measuring the thermal conductivity with a LECO Truman N analyser (Leco Corporation, St. Joseph, Michigan). The other elements (P, K, Ca, Mg) were quantified using inductively coupled plasma atomic emission spectroscopy (model ICAP 9000, Thermo Instruments, Franklin, Massachusetts). The mineral content (concentration ^∗^ dry mass) of each element was calculated, thus reflecting plant nutrient uptake and accumulation (Timmer et al., 1991). Because the tissues were grouped in composite samples for analysis, the mineral content was calculated for an average tissue mass. Substrate fertility (N-NO_3_, N-NH_4_, N_min_, P, K, Ca, Mg) was also analyzed for composite substrate samples collected from the root plug of seedlings from the same orchard and block. The chemical analyses were conducted by the QMFWP’s Laboratoire de chimie organique et inorganique (certified ISO/CEI 17025).

### Gas Exchange Measurements for Different Seed Sources (2+0)

Gas exchange measurements were conducted on seedlings during their second growing season (2+0). On August 3, 2012, one seedling/orchard/block was randomly sampled at the St-Modeste forest nursery, for a total of 40 seedlings. The seedlings were placed in a growth chamber at Université Laval and measurements were taken between August 6 and 8, 2012 using a LI-COR 6400 (LI-COR Inc., Lincoln, NE, USA). Light intensity in the growth chamber was approximately 800 μmol/m^2^/s, whereas temperature was 20°C and relative air humidity 55%. Measurements were taken on a section of a lateral branch representing the second year of growth. This branch had to be lignified and the needles had to be fully developed. To avoid water stress, the seedlings were irrigated and saturated by capillarity by placing the seedling containers in a bin of water before the measurements. Three variables were measured: net photosynthesis (A), transpiration (E) and stomatal conductance (g_sw_). The measurements were taken at ambient atmospheric CO_2_ partial pressure (Ca = 380 μmol mol^-1^) and at saturated photosynthetic active radiation (PAR = 800 μmol m^-2^ s^-1^). Within the leaf cuvette, air temperature was maintained at 20°C, while vapor pressure deficit and RH were fixed at 1.0 kPa and 55, respectively. Three consecutive stable measurements were averaged to obtain the estimate of each gas exchange variable. Water use efficiency was calculated using the following formula:

(1) $$WUE\;=\;\frac{A}{g_{sw}}$$

Following gas exchange measurements, the branch was excised and immersed in liquid nitrogen. All of the needles were then detached from the branch for calculation of the projected foliar surface area using the WinSeedle software (Regent Instrument Inc., Québec, QC, Canada).

### Climatic Data for the First Generation Seed Orchards

As each of the first-generation seed orchard is composed of trees selected from local natural stands, 30-year average (1981-2010) climatic data estimates were obtained using the centroid of the plus-trees selected to make them up, using the BioSIM software (version 10.2.5.39) (Régnière and Saint-Amant, 2008). The estimates were obtained by interpolation of temperature and precipitation data collected in the eight closest weather stations of the orchard and adjusted for differences in latitude, longitude and elevation (Régnière and Saint-Amant, 2008). This method could not be used to estimate climatic data for the second-generation seed orchards because they are composed of trees of many different origins from the provinces of Québec and Ontario; moreover, temperature and precipitation “mean provenances values” would not be representative of the climatic conditions of the orchards sites, and would not permit the determination of accurate correlations between performance and climatic conditions.

### Planting Sites: Simulating Assisted Migration Scenarios

After two growing seasons in forest nursery (2+0), seedlings of the same seedlots were planted on three planting forest sites in eastern Québec, covering three bioclimatic domains (**Figure 1**). The average seedling characteristics of each seed orchard were assessed before planting. Average values for height (33.5 cm-41.9 cm), total mass (11.9 g–15.5 g) and shoot nitrogen content (1.5%-1.68%) were calculated from a sample of 15 seedlings/orchard. The field tests were established on May 28-29, 2013 in Watford township in the Beauce region (46.3°N 70.4°W, Sugar maple- yellow birch domain), June 4-6, 2013, in Asselin township in the Témiscouata valley (47.84°N 68.52°W, Balsam fir yellow birch domain) and June 11-13 in Deville township on the Gaspé peninsula (48.62°N 65.72°W, Balsam fir-white birch domain). The first site (Watford) simulates a transfer to the southern limit of the optimal continuous distribution zone of white spruce, which is a minor component of forest stands (depending on the location, white spruce represents between 0 to 7% of the forest cover in the maple domain). The second (Asselin) and third (Deville) sites simulate seed source transfer inside the continuous natural distribution of the species (between 7 and 30% of the forest cover in these domains is comprised of white spruce). Textural analyses of the soil revealed the presence of loamy soils for the Watford and Asselin sites and a clay-loam soil in Deville. For each site, four complete blocks were established with the eight seed sources randomly distributed in 27 m ^∗^ 27 m plots with 144 plants/plot. Seedlings were planted at density of 2000 seedlings/ha as recommended for white spruce (Bureau du forestier en chef, 2013). Vegetative competition was removed in mid-July, 2013.

At the end of the first growing season, the height of 64 interior seedlings/orchard/block was measured on each of the three sites.

### Statistical Analyses

Analyses of variance were conducted for each trait using the MIXED procedure (SAS Institute Inc., 2011). A repeated-measures model was used for the variables that were measured bi-weekly (height, diameter, dry mass and tissue mineral nutrition). In this case, sampling dates and orchards were considered to be fixed effects and blocks to be random effects. The most appropriate variance/covariance structure was used for each of the analyzed traits to take into account the autocorrelation between the measurements made on the same experimental units. Several structures were tested and the final selection was made to minimize the likelihood value and the number of parameters. The assumptions of normality and homogeneity of variance of the residuals were verified using SAS UNIVARIATE procedure. The level of significance was set at *P* < 0.05. When the interaction between the effect of orchard and that of sampling date was found to be significant, the comparisons between orchards were made for the last sampling date for each of the two growing seasons (1+0 and 2+0). Multiple comparisons between means were conducted using simulations (Westfall et al., 1999) to determine the source of the differences. The comparisons of means for the gas exchange traits (A, g_sw_, WUE, and E), were made using the Tukey test.

To determine similarities between orchards with regard to the different morpho-physiological traits, a cluster analysis was performed for the last sampling date of the second growing season (2+0) using the Ward method of the CLUSTER procedure. The grouping was performed using three traits (total height, root dry mass, and shoot nitrogen content) indicative of seedling performance and used to evaluate seedling quality for reforestation in Québec (Veilleux, 2013). The average values for these three traits were standardized before cluster analysis in order to obtain comparable scales. The number of groups retained was based on *R*^2^ value, which reflects the differences between clusters.

Growth curves for height during the second growing season for each of the orchard groups were fit to an asymptotic logistical model using the NLMIXED procedure (SAS Institute Inc., 2011):

(2) $$Y\;=\;\frac{a}{1\;+\;e^{-c(day-b)}}\;+\;u\;+\;\varepsilon$$

where Y is the seedling height on a given date; a, the final height at the end of the second growing season (asymptote of the curve); b, the date at which half of the final height is attained (the point of inflection of the curve); c, the component of the growth rate; e, the base of the natural logarithm; u, the random effect of block and; *𝜀*, the residual error. The date of the final height corresponds to the date of growth cessation and is commonly used as an estimate of bud formation.

The curve parameters for each group of orchards were compared using the Bonferroni test. The level at which differences were considered significant corresponded to the retained level of significance (*P* < 0.05) divided by the number of 2 × 2 comparisons required for each parameter.

A multiple regression analysis between final height (2+0) of the seedlings of the six first-generation seed orchards and the climatic conditions at the orchards location was also carried out. Ten climatic variables (growing season temperature, average annual temperature, maximum temperature, minimum temperature, average temperature during the month of July, degree-days ≥5°C, growing season precipitation, total annual precipitation, aridity index and length of growing season) were used. The RSQUARE option of the REG procedure was employed to determine the combination of variables producing the highest coefficient of determination (*R*^2^). The effect of collinearity was verified using the TOL, VIF, and COLLIN options of the same procedure.

For the seedling height measured under three different site conditions, the analysis of variance was conducted using the MIXED procedure. Site, orchard and the site^∗^orchard interaction were considered to be fixed effects, whereas block was considered to be random effect. Assumptions of normality and homogeneity of variance of the residuals were verified and the means were compared using a Tukey test (*P* < 0.05).

## Results

### Growth Variables of White Spruce Seedlings (1+0 and 2+0) in the Nursery

Analyses of variance showed significant effects of orchard (*P* < 0.001) for all of the growth traits measured during the first growing season except for root dry mass (*P* = 0.09). The effect of sampling date was also significant for all of the traits measured. The date^∗^orchard interaction was significant for the total dry mass (*P* = 0.04) and the shoot dry mass (*P* = 0.01) only. At the end of the first growing season (1+0)(October 26, 2011), the comparisons of means of shoot dry mass and total dry mass showed no significant differences among seedlings from different orchards. The average height attained was 9.9 ± 1.9 cm whereas the average diameter was 2.7 ± 0.5 mm. The average dry masses were 0.93 ± 0.29 g for shoots, 0.38 ± 0.12 g for roots, and 1.31 ± 0.39 g for the entire seedling.

**FIGURE 2:**
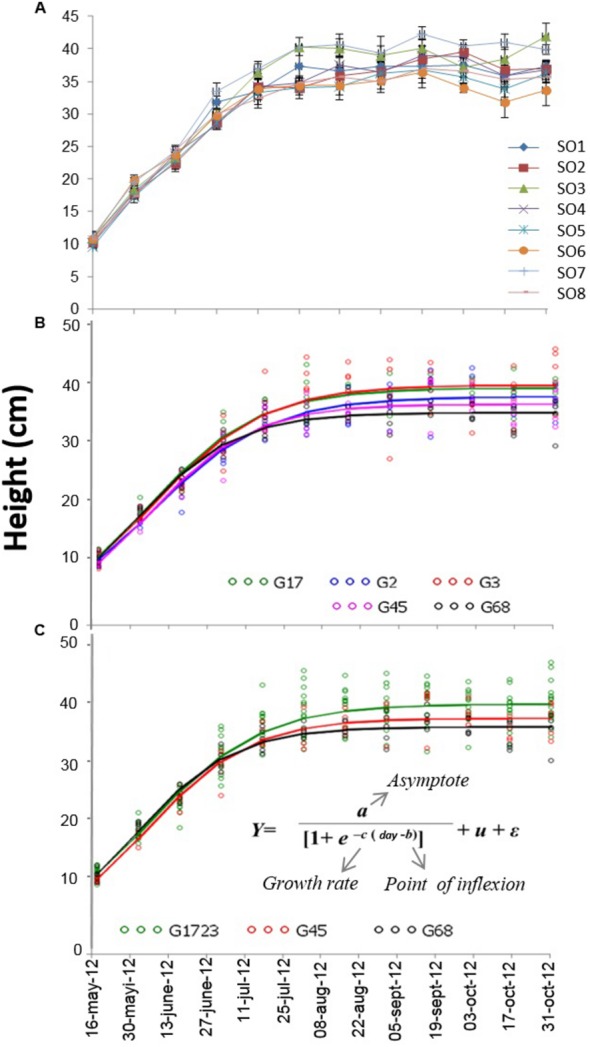
**(A)** Growth curves for height of white spruce seedlings from eight seed orchards (SO1 to SO8) during their second growing season (2+0) in a forest tree nursery. Measurements were taken bi-weekly (*n* = 25). **(B)** Height growth curves for the five orchard groups (G17 [group comprised of SO1 and SO7], G2, G3, G45 [group comprised of SO4 and S05], and G68 [group comprised of SO6 and SO8]) modeled using a logistical growth model. The orchards were grouped with respect to three seedling growth traits measured at the end of the second growing season (2+0). **(C)** Height growth curves for the seedlings of three orchard groups (G1723, G45, and G68) modeled using a logistical growth model. The orchards were grouped from the five groups that had previously been assembled based on similar growth parameters of different fitted logistical models (see text).

During the 2+0 growing season, the effects of orchard and date were significant for all of the growth traits (diameter *P* < 0.001), shoot dry mass (*P* < 0.001), root dry mass (*P* = 0.01), total dry mass (*P* = 0.002)), whereas the date^∗^orchard interaction was only significant for height (*P* = 0.02). The comparisons made at the end of the second growing season (October 17, 2012) revealed that seedlings from SO3 attained a height (41.9 ± 6.7 cm) that was higher than those of SO6 (33.6 ± 6.8 cm) and SO8 (35.7 ± 5.1 cm), and that seedlings from SO7 were taller (39.8 ± 5.9 cm) than those of SO6 (33.6 ± 6.8 cm) (**Figure 2A**).

### Mineral Nutrition

During the first growing season (1+0), the effects of orchard and date were significant for all of the mineral elements (N, P, K, Ca, Mg) contained in the shoot tissues and whole seedlings, whereas the effect of orchard was not significant for K content (*P* = 0.2). The date^∗^orchard interaction was only significant for K content of the shoots (*P* = 0.0005) and whole seedlings (*P* = 0.004). The seedlings from SO2 had a higher (14.16 ± 1.13 mg) K content than those from SO4 (7.47 ± 1.13 mg), SO5 (8.42 ± 2.39 mg), and SO8 (8.58 ± 2.50 mg). The K content of shoots from SO3 (12.28 ± 2.08 mg) was greater than those from SO4 (7.47 ± 2.08 mg). The effect of orchard was significant for all of the other mineral elements (N, P, Ca, Mg) in the shoot tissues as well as for the K content of the roots. With respect to mineral concentrations of the shoot tissues, the date^∗^orchard interaction was significant for N (*P* = 0.02) and K (*P* = 0.04). Comparisons of means revealed that SO4 (2.61 ± 0.12%), SO6 (2.61 ± 0.11%) and SO7 (2.60 ± 0.09%) attained higher shoot N concentrations than SO2 (2.32 ± 0.19%). In the case of K, the seedlings from SO2 (1.34 ± 0.15%) had a higher concentration than those from SO4 (0.86 ± 0.09 %) and SO7 (1.00 ± 0.14%), and the concentration in seedlings from SO3 (1.19 ± 0.19%) was higher than those from SO4 (0.86 ± 0.09%).

During the second growing season (2+0), the effect of orchard was significant for all of the mineral elements in the shoot tissue, whereas it only significantly affected the N content in the root tissue (*P* = 0.04) and the K content of the whole seedlings (*P* = 0.01). The effect of date was significant for all of the mineral elements and for all types of tissues. However, the date^∗^orchard interaction was only significant for K content of the shoots (*P* = 0.03) and whole seedlings (*P* = 0.04). However, the comparisons of the contents on the last sampling date (October 17, 2012) showed no difference among seedlings from the different orchards.

### Gas Exchange and Water Use Efficiency Variables

**FIGURE 3:**
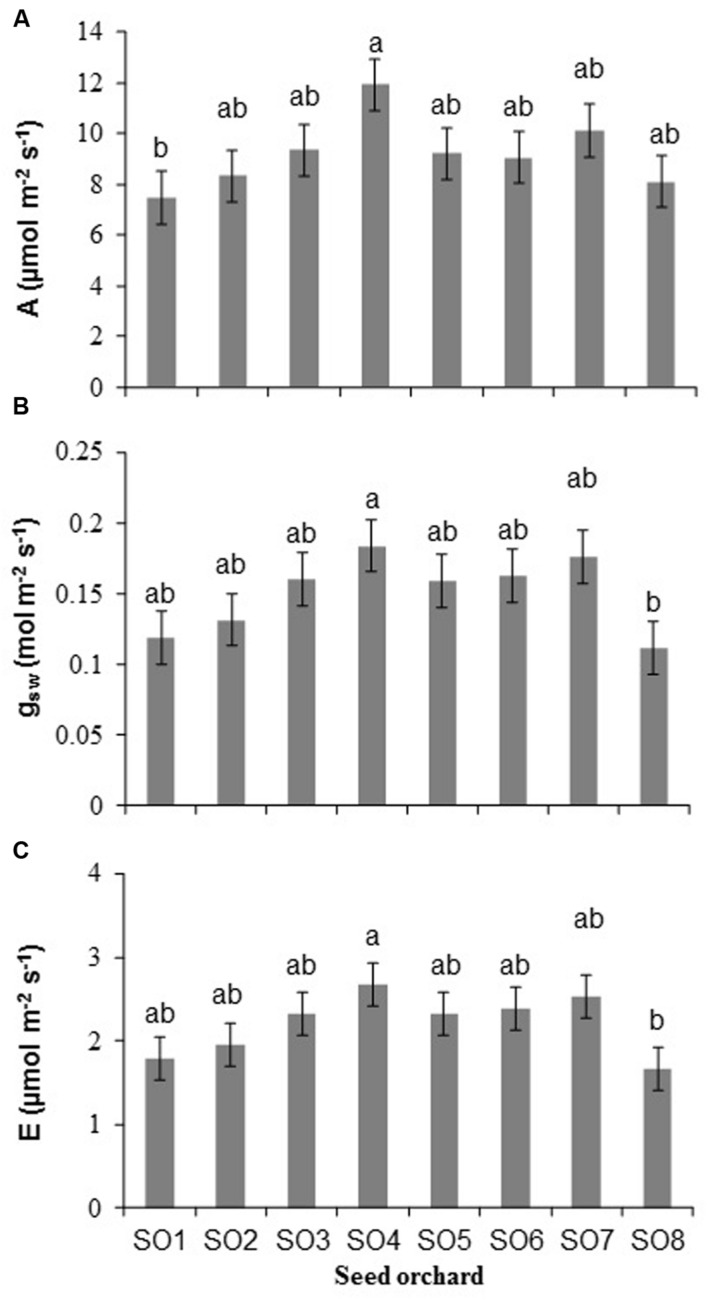
**(A)** Rate of net photosynthesis, **(B)** stomatal conductance, and **(C)** transpiration of white spruce seedlings from eight seed orchards (SO1 to SO8). Measurements made in August of the second growing season (2+0) (mean and standard error, *n* = 5 seedlings). For a given variable, averages having the same letter are not significantly different according to a Tukey test (α = 0.05).

Significant differences among orchards were observed for net photosynthesis (A) (*P* = 0.03), stomatal conductance (g_sw_) (*P* = 0.03), and transpiration (E) (*P* = 0.04) (**Figure 3**). The rate of photosynthesis of seedlings from SO4 was higher than those from SO1 (**Figure 3A**). The stomatal conductance and transpiration of seedlings from SO4 were higher than those from SO8 (**Figures 3B,C**). No significant difference (*P* = 0.26) in water use efficiency (WUE) was observed among orchards.

### Grouping of Orchards and Height Growth Modeling

**FIGURE 4:**
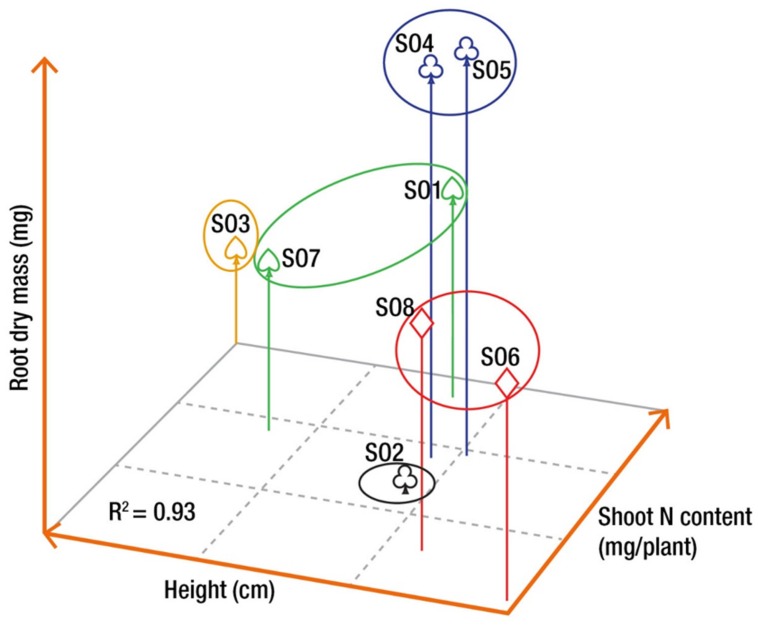
**Grouping of the seed orchards based on three morphological variables measured on seedlings at the end of the second growing season in nursery, i.e., root dry mass, height, and shoot nitrogen (N) content.** The grouping was achieved using the Ward method of the Cluster procedure of SAS (see Materials and Methods). Orchards with similar symbols are part of the same group.

Cluster analysis based on three traits selected a priori (height, root dry mass, and shoot nitrogen content) measured at the end of the second growing season (October 17, 2012) resulted in five distinct groups of orchards, explaining 93% of the total variance (**Figure 4**). The first group was composed of SO1 and SO7 (G17), whereas SO2 (G2) and SO3 (G3) did not cluster with any other seed orchard. SO4 and SO5 (G45) formed the forth group and SO6 and SO8 (G68) made up the fifth group (**Figure 4**).

**FIGURE 5:**
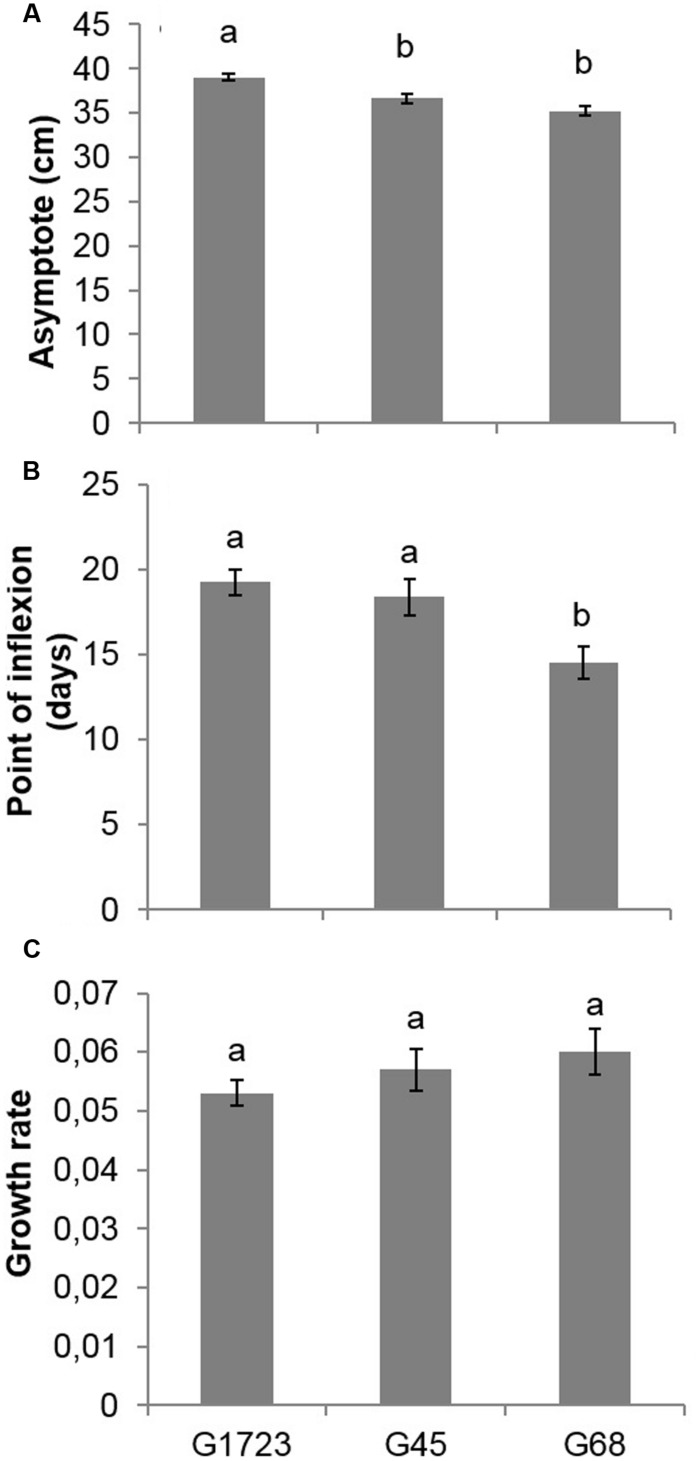
**(A)** Asymptote, **(B)** point of inflection, and **(C)** growth rate (mean and standard error) of the three height growth curves modeled using a logistical growth model for the second growing season (2+0) of white spruce seedlings in a nursery for the three delimited groups of white spruce seed orchards (G1723, G45, G68) (see **Figure 2B** and text). For example, G45 means the group consisting of seedlings from seed orchards SO4 and SO5. For a given variable, means having the same letter are not significantly different according to a Bonferroni test (α = 0.017).

The comparisons of parameter *a* (asymptote of the curve), following the modeling of the height growth curves of each group (**Figures 2A,B**), indicated that G17, G2, and G3 had similar parameters, yet significantly larger than those of groups G45 and G68. The comparisons of parameter *b* (inflection point of the curve) indicated that the G68 group was significantly different from the other four groups of seed orchards. Finally, the comparison of parameter *c* (growth rate) indicated that all groups were similar (results not presented). Given that SO1, SO7, SO2, and SO3 had similar height growth curves, it was decided to group these orchards together and model the growth curves of the newly established groups of orchards, i.e., the groups comprised of SO1, SO7, SO2, and SO3 (G1723), SO4 and SO5 (G45) and SO6 and SO8 (G68) (**Figures 2B,C**). Comparisons of parameters resulting from this modeling (**Figure 5**) show that the G1723 group was significantly taller (*a* = 39.1 cm) than groups G45 (*a* = 36.7 cm) and G68 (*a* = 35.2 cm) (**Figure 5A**). However, group G68 reached its final height significantly faster (*b* = 15 days) than groups G1723 (*b* = 19 days) and G45 (*b* = 18 days) (**Figure 5B**) Even though the comparisons of parameter *c* indicated that the height growth curves for the three orchard groups had the same growth rate (**Figure 5C**), the three groups showed distinct growth patterns (**Figures 2C** and **5A,B**).

### Performance with Respect to Orchard Climate

Among the ten climatic variables tested in the different models, a combination of three variables, i.e., mean growing season temperature, average temperature during the month of July and length of growing season, were retained, while avoiding the elevated effects of collinearity. This three-variable model explained 30% (*R*^2^*-adjusted* = 0.30) of the variance for the final height of the (2+0) seedlings (*P* = 0.01):

(3) $$H=47.31512-40.66226^{*}Tm+45.84888^{*}Tj-1.62881^{*}Ls$$

where *H* is the height in centimeters; *Tm*, the average growing season temperature in degrees; *Tj*, the average July temperature in degrees; and *Ls*, the length of the growing season in days.

### Seedling Growth during Their Establishment Phase on the Planting Sites

**FIGURE 6:**
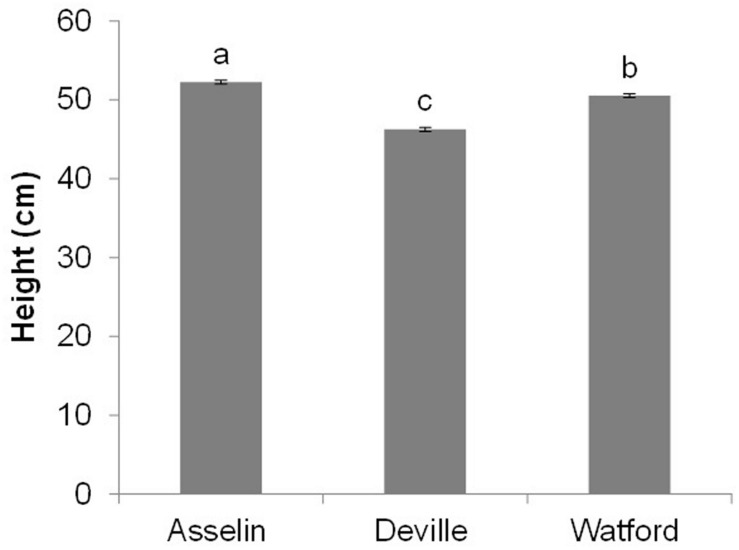
**Overall average height (mean and standard errors) of white spruce seedlings from eight seed orchards (SO1 to SO8) at the end of the first growing season on three reforestation sites (mean and standard error, *n* = 2048 seedlings per site).** Means having the same letter are not significantly different according to a Tukey test (α = 0.05).

**FIGURE 7:**
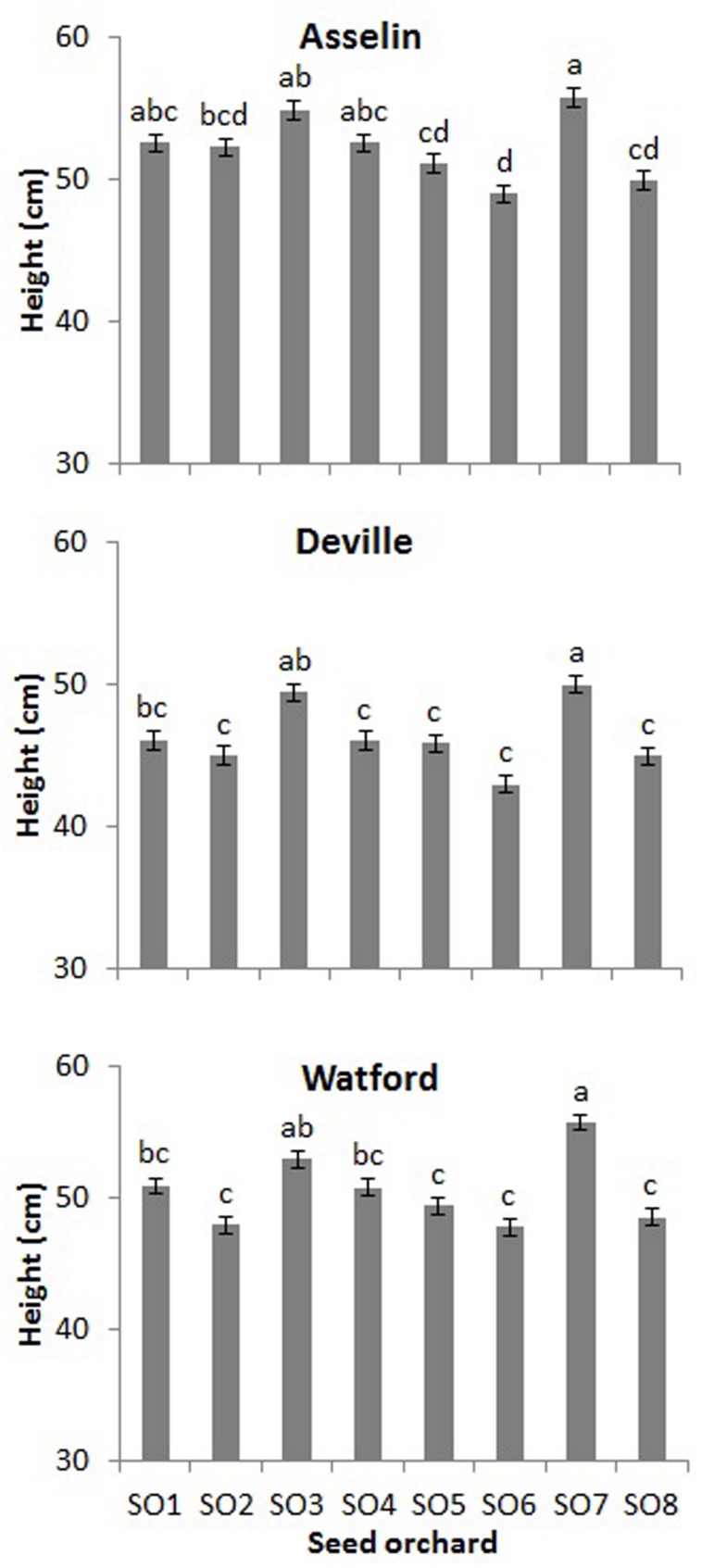
**Height (mean and standard errors) of white spruce seedlings from each of the eight seed orchards (SO1 to SO8) for the three forest plantation sites (Asselin, Deville, and Watford) at the end of the first year of establishment (2+1) (mean and standard error, *n* = 256 seedlings per seed orchard).** Means having the same letter are not significantly different according to a Tukey test (α = 0.05).

After the first growing season on the three planting sites (2+1), seedling height was significantly affected by site and orchard (*P* < 0.001), whereas the interaction of site^∗^orchard was not significant (*P* = 0.29). Seedlings on the Asselin site were significantly taller (52.2 cm) than those growing on the most south-westerly site, Watford (50.2 cm), and the most north-easterly site, Deville (46.3 cm) (**Figure 6**). Seedlings from SO3 and SO7 were, on average, taller than seedlings from other orchards, and seedlings from SO6 and SO8 were shorter, regardless of plantation site (**Figure 7**). The survival rate of seedlings was similar among the seed orchards and the sites, and was greater than 98%.

## Discussion

By evaluating the functional traits of seedlings from different genetic seed sources used in Québec in different growing conditions, the aim of this study was to help build the scientific foundation for operational seed source transfer in the context of climate change, also called assisted migration. This approach combined the assessment of morpho-physiological responses of different white spruce seed sources in both forest nurseries located in the natural range of the species, and on different planting sites that simulate various climatic transfers of seed sources. The evaluation of these responses at an early stage is also motivated by the fact that the establishment phase of future plantations and forests represents a critical step (Grossnickle, 2000).

The evaluation of growth and mineral nutrition traits of white spruce seedlings revealed the presence of significant morpho-physiological differences among the different seed sources. The use of relatively optimal and uniform conditions (environmental factors, irrigation, fertilization, growing substrate, etc.) in the nursery was meant to foster the expression of genetic potential, indicating various levels of genetic adaptation among the different seed sources analyzed.

### Grouping of Seed Orchards

Assessment of seedling morphological traits made it possible to identify three groups of seed orchards based on three traits selected a priori, namely final height, root mass, and nitrogen content (**Figure 4**). These groups also showed different trends of height growth during the growing season (**Figure 2**). The height growth curves modeled for each of the orchard groups (**Figure 2C**) showed that southern seed sources (SO1 and SO3) and those from the second-generation orchards (SO2 and SO7) had higher height growth relative to sources from the west (SO4 and SO5) and the north-east (SO6 and SO8) (**Figure 5A**). Our results agree with those obtained by Lesser and Parker (2004) in a study of young white spruce seedlings grown from 127 seed sources from Ontario and Quebec which showed that, in general, the southerly seed sources had a greater capacity for height growth at the end of the second growing season. A north-south cline was also observed in 63 young white spruce populations from Ontario and Quebec grown under nursery conditions (Li et al., 1997) and in a two-year nursery study of ten first-generation Quebec seed orchards (Carles et al., 2011), indicating local adaptation to temperature. In a study involving 8- and 13-year-old white spruce trees from 26 seed sources, representing six ecological regions of the temperate and boreal forest in Quebec, Jaramillo-Correa et al. (2001) also showed that populations from southern ecological regions grew taller than those from northern regions. Such a cline following temperature variation was also observed for black spruce (*Picea mariana*) in the area of sympatry with white spruce in Quebec (Beaulieu et al., 2004), and it had been observed for other conifers in other parts of the world, such as *Pinus patula* (Saenz-Romero et al., 2011) and *Pinus pseudostrobus* (Saenz-Romero et al., 2012) in Mexico, where quantitative trait variation following altitude and temperature variation was observed in controlled environment tests for both species. It was also recently shown, at least for the northeastern part of the natural range of white spruce in North America, that most of adaptive genetic variation in genes significantly associated to climate was also related to temperature, with many significant linear relationships noted between allelic variation and temperature, which is mainly following a north-south latitudinal trend in this region (Hornoy et al., 2015).

The fact that the two second-generation seed orchards (SO2 and SO7) were regrouped is not surprising, given that a large portion (40%) of families from which the plus-trees were selected are common to both seed orchards. Previous results have indeed shown that many open-pollinated families performed well in genetic tests established both in the sugar maple and the balsam fir bioclimatic domains (Beaulieu, 1996; Li et al., 1997).

The results of the cluster analysis also seem to imply that seed zones could be merged. For instance, SO4 and SO5 could likely supply seed for a large area in northwest Québec. Likely, SO6 and SO8 could supply seed for the reforestation of the Gaspé Peninsula and the St. Lawrence North Shore region. Finally, all the southern parts of the province of Québec covered by the sugar maple bioclimatic domain could likely be supplied by both SO1 and SO3, but also by the second-generation seed orchard SO2. These results are, however, too early to make final decision about the merging of seed zones, but they constitute a solid basis for potential future assisted migration decisions. These results are also in agreement with the early findings of Li et al. (1997) for white spruce in Québec where a small number of genetically distinct seed zones was delimited.

### Mineral Nutrition and Gas Exchange Variables

At the end of the second growing season (2+0), the mean foliar nitrogen for the seedlings from the eight orchards (*N* = 1.6 ± 0.1 %) was within the required range of 1.4 to 2.2% nitrogen, to ensure the survival and good growth of containerized conifer seedlings (Landis, 1989). It has been shown that, for large size white spruce seedlings, a foliar nitrogen concentration higher than 1.5% results in adequate net photosynthetic rates and good root regeneration, thus allowing adequate growth and survival rates after outplanting (Gagnon and Lamhamedi, 2011). Although there was no difference in net photosynthesis rates among southern and northern seed sources, as observed by Bigras and Bertrand (2006) for black spruce, the overall mean for all of the orchards combined was 9.2 μmol m^-2^ s^-1^, which is comparable to values of between 6 and 15 μmol m^-2^ s^-1^ obtained in several other studies involving white spruce (Awada and Redmann, 2000; Grossnickle, 2000; Sullivan and Sveinbjornsson, 2011).

### Bud Formation

Our results, obtained under uniform environmental conditions of temperature, precipitation and photoperiod in the nursery, revealed that seedlings from north-eastern seed sources (SO6 and SO8), which exhibited smaller growth than other sources, set their buds four days earlier than those from southern and second generation orchards (**Figure 5A**), resulting in an earlier cessation of height growth in seedlings from northeastern sources. Furthermore, from a study of 3-year old nursery grown white spruce seedlings from 57 provenances in Québec and Ontario, it has been reported that taller seedlings entered dormancy later, on average, than shorter ones (Li et al., 1993). The same relationship was observed for 30 Québec black spruce seed sources each represented by three half-sib families for height growth and date of bud set assessments taken over several years on two forest plantation sites (Prunier et al., 2013). Thus, for spruces in Québec, these results indicate that the growing season is generally longer for seed sources displaying higher growth, which are those generally originating from more southern latitudes. It implies that these sources are genetically conditioned to exploit better the more favorable growing conditions encountered under these latitudes. It suggests that under warming conditions, these seed sources would be better adapted than local ones, as also inferred by Andalo et al. (2005) from assessing white spruce tree height and diameter at 22 years of age.

### Adaptation of Seed Sources to Climate of Origin

For first-generation seed orchard seedlings, we observed that height at the end of the second growing season was related to three local growing season temperature variables characterizing the origin of the various seed sources. Height was negatively correlated with the mean growing season temperature and length of growing season, but positively correlated to the mean temperature of the month of July. Contrary to results previously observed for 45 white spruce seed sources in Québec used for modeling the outcomes of four climate change scenarios (Andalo et al., 2005), the performance of the present seed sources was not linked to precipitation variables. This could be due to a larger range of longitude sampled in the previous study, which extended westward to Ontario. Our results are also in line with those of Hornoy et al. (2015), who reported much fewer significant correlations between gene allelic diversity and precipitation than with temperature among white spruce natural populations mostly from Québec. At the same time, the adaptation of the seed sources to the local climatic conditions of their origin may have favored the growth of seed sources originating from orchards in proximity of the nursery where the study was conducted. This could partially explain the inferior height growth of white spruce seedlings from orchards in the west and north-east of Québec, where the climate is cooler. For this reason, it would be appropriate to repeat the study by characterizing the same seed sources in nurseries located in northern and/or western Québec.

### Performance of the Seed Sources during the Establishment Phase

The results obtained during the establishment phase, that is, at the end of the first growing season on the reforestation sites, revealed a very high survival rate and complete absence of frost damage on the three different planting sites. Though it must be validated on multiple years, this trend suggests adequate acclimatization and adaptation of the different seed sources to the climatic conditions encountered in the three bioclimatic domains. The absence of observed mortality on the three planting sites during the establishment phase is likely related to the high phenotypic plasticity of white spruce (Loo et al., 2015). Similar results were observed for 52 white spruce somatic clones following their transfer to two planting sites in the sugar maple and balsam fir domains (Wahid et al., 2012b) and for 57 white spruce provenances from Québec and Ontario planted on three sites in the sugar maple and balsam fir domains where the performance of 3-year old seedlings was stable regardless of planting site, 5 years after outplanting (Li et al., 1993).

The assessment of seedlings height during the establishment phase on forest sites indicated the presence of a significant variation among seed sources. Height growth, like hardening and bud set, is a trait that usually shows moderate, but significant, heritability (*h*^2^_F_ = 0.17 to 0.45) in white spruce families, for instance under nursery growth conditions and when measured 8 year-old trees on forest planting sites (Li et al., 1993; see also Cornelius, 1994; Wahid et al., 2013). Heritability for height was stronger for clones measured during their first 2 years of growth in a nursery (*H^2^_C_* = 0.60) (Wahid et al., 2012b, 2013) and after four years of clonal tests (*H^2^_C_* = 0.26) (Wahid et al., 2012b), as well as for white spruce rooted cuttings from 75 families at the end of their first (*h^2^_F_* = 0.76) and second (*h^2^_F_* = 079) growing seasons (Gravel-Grenier et al., 2011).

The transfer of forest tree seed sources has been practiced for a long time (Wright, 1976). But the use of this approach as an operational means of adapting plantations to climate change is a concept that has attracted much attention over the past 10 years (Hewitt et al., 2011) and even before, where planting programs of non-local seed sources in this context had already been advocated in the early 1990s (Ledig and Kitzmiller, 1992). At the international scale, several research projects have been conducted or are presently underway to take advantage of intraspecific variation in order to better face the anticipated effects of climate change: by evaluating the effect of transferring genetic resources within the limits of species natural distributions (O’Neill et al., 2008; Thomson and Parker, 2008), but also for testing the movement of species over long distances beyond their current natural distributions. For instance, in Canada, a vast project involving 16 species from British Columbia, and the states of Washington, Oregon and Idaho was launched to assess the performance of seed sources transferred to high latitudes in the Yukon as well as study the tolerance of northern sources to global warming by planting them at lower latitudes in California (Marris, 2009; Williams and Dumroese, 2013b).

The use of seeds at an operational scale in an assisted migration program requires extensive knowledge of the genetic material both in controlled and in natural environments (Williams and Dumroese, 2013a). Our previous results (Benomar et al., 2015, 2016) and the findings of the present study are very encouraging and indicate that current seed orchard zones could likely be merged as a first step to face climate change challenges. In the long-term, the evaluation of the morphological responses of different spruce seed sources *in situ* on numerous planting sites and for multiple traits related to adaptation (growth, tolerance to biotic and abiotic stresses such as frost, drought, susceptibility to forest pests, etc.) should help refine the current seed transfer models which are generally established based on empirical models (Beaulieu et al., 2004; Andalo et al., 2005; Rainville et al., 2014).

## Conclusion

The morpho-physiological characterization of white spruce seedlings from eight seed sources both under nursery conditions during their first and second growing seasons (1+0 and 2+0) and after 1 year of growth on three reforestation sites allowed for a rapid evaluation of their growth potential during the juvenile stage, which is critical for their successful establishment. In addition to permitting an early selection of genetic material based on a combination of observed performance in the nursery and on forest planting sites, the originality of our results resides in the illustration, for first-generation seed orchards, of the significant relationship between height at the end of the second growing season and the local climate at the site of seed orchard origin, notably the average growing season temperature, temperature during the month of July and the length of the growing season. Given that these seed orchards were each made up of genetic selections in local natural populations, in order to meet local needs of improved seed, these correlations suggest that these climatic parameters could be taken into consideration when choosing reforestation sites in order to match genetic potential and facilitate seedling adaptation. The different morpho-physiological responses observed in the relatively controlled nursery environment are likely attributable to the expression of the full genetic potential of the different seed sources. Combined with transfer models, these differences in morpho-physiological responses will help define new seed transfer zones that should help ensure the adaptation of white spruce plantations in the context of climate change in Québec.

The approach of the present study, which combines nursery and forest planting site growth assessments, has practical implications for reforestation. The medium and long-term evaluations of different seed sources on different plantation sites will contribute to the selection of best adapted seed sources, depending on the amplitude of the transfer (within, at the margin of the natural distribution of the species, or over longer distances). It will help optimize the implementation of the choice of genetic resources in the context of assisted migration by maximizing, over the short term, the compatibility between the climate observed at the location of origin of the seed sources and the choice of potential planting sites based on the climate variables that were revealed to be the most significant in the present study. It will also help identify the seed sources (e.g., orchards and breeding populations) that are best adapted to current and future climatic conditions. The combination of these different results will further help clarify the ecological amplitude of use for each seed orchard and generate maps for transfer and suitability of reforestation sites for each seed source.

It also appears to be important to pursue a medium-term evaluation, in space and time, of the productivity of plantations made up of seedlings from diverse seed sources in relation to variations in climate (temperature, precipitations, length of growing season, etc.) and to evaluate the morpho-physiological reaction of different seed sources in response to the multiple interactions with different sources of environmental stress. This information will contribute, over the long term, to assess whether the adopted assisted mitigation strategy, based on knowledge acquired through morpho-physiological research, has been effective.

## Author Contributions

This paper is taken from IV M.Sc. under the official direction of HM and MSL. HM, MSL, JBe, JBo, AR, and LB conceived the study and obtained the funding. All authors participated in the drafting of the manuscript. AR participated to designing the genetic tests and providing the seed. MSL and his team have installed three weather stations for the acquisition of environmental variables and conducted mineral analyses. IV performed the measurements of different variables, the analysis and the interpretation of data. All authors read and approved the final version of the manuscript.

## Conflict of Interest Statement

The authors declare that the research was conducted in the absence of any commercial or financial relationships that could be construed as a potential conflict of interest.
